# The xenobiotic transporter *ABCC4*/MRP4 promotes epithelial mesenchymal transition in pancreatic cancer

**DOI:** 10.3389/fphar.2024.1432851

**Published:** 2024-07-24

**Authors:** S. N. Gancedo, A. Sahores, N. Gómez, N. Di Siervi, M. May, A. Yaneff, M. G. de Sousa Serro, N. Fraunhoffer, N. Dusetti, J. Iovanna, C. Shayo, C. A. Davio, B. González

**Affiliations:** ^1^ Instituto de Investigaciones Farmacológicas (Universidad de Buenos Aires-Consejo Nacional de Investigaciones Científicas y Técnicas), Ciudad Autónoma de Buenos Aires, Buenos Aires, Argentina; ^2^ Programa Franco-argentino de Estudio del Cáncer de Páncreas, Buenos Aires, Argentina; ^3^ Centre de Recherche en Cancérologie de Marseille (CRCM), INSERM, CNRS UMR, Aix-Marseille Université and Institut Paoli-Calmettes, Parc Scientifique et Technologique de Luminy, Marseille, France; ^4^ Equipe Labellisée La Ligue, Marseille, France; ^5^ Hospital de Alta Complejidad El Cruce, Argentina. Universidad Nacional Arturo Jauretche, Buenos Aires, Argentina; ^6^ Instituto de Biología y Medicina Experimental (Consejo Nacional de Investigaciones Científicas y Técnicas), Buenos Aires, Argentina

**Keywords:** PDAC, ABCC4/MRP4, epithelial mesenchymal transition, prognosis marker, FOXA1 GATA2

## Abstract

The xenobiotic transporter *ABCC4*/MRP4 is highly expressed in pancreatic ductal adenocarcinoma (PDAC) and correlates with a more aggressive phenotype and metastatic propensity. Here, we show that *ABCC4* promotes epithelial-mesenchymal transition (EMT) in PDAC, a hallmark process involving the acquisition of mesenchymal traits by epithelial cells, enhanced cell motility, and chemoresistance. Modulation of *ABCC4* levels in PANC-1 and BxPC-3 cell lines resulted in the dysregulation of genes present in the EMT signature. Bioinformatic analysis on several cohorts including tumor samples, primary patient-derived cultured cells, patient-derived xenografts, and cell lines, revealed a positive correlation between *ABCC4* expression and EMT markers. We also characterized the *ABCC4 cis*trome and identified four candidate clusters in the distal promoter and intron one that showed differential binding of pro-epithelial FOXA1 and pro-mesenchymal GATA2 transcription factors in low *ABCC4*-expressing HPAF-II and high *ABCC4*-expressing PANC-1 xenografts. HPAF-II xenografts showed exclusive binding of FOXA1, and PANC-1 xenografts exclusive binding of GATA2, at *ABCC4* clusters, consistent with their low and high EMT phenotype respectively. Our results underscore *ABCC4*/MRP4 as a valuable prognostic marker and a potential therapeutic target to treat PDAC subtypes with prominent EMT features, such as the basal-like/squamous subtype, characterized by worse prognosis and no effective therapies.

## Introduction

Pancreatic ductal adenocarcinoma (PDAC) represents one of the greatest therapeutic challenges in modern oncology due to its highly aggressive nature, dismal prognosis, and resistance to current therapies. It is projected to become the second leading cause of cancer-related mortality by 2030, with limited therapeutic advancements in recent years (Kenner et al., 2016; Sarantis et al., 2020). Emerging evidence implies the epithelial to mesenchymal transition (EMT) as a pivotal process driving PDAC progression, metastasis, and treatment resistance (Palamaris et al., 2021). EMT involves the reversible transformation of epithelial cells into mesenchymal-like cells, fostering traits associated with loss of intercellular contacts, enhanced cell migration, intratumoral heterogeneity, drug tolerance, and stemness (Palamaris et al., 2021). The orchestration of EMT transcriptional reprogramming is controlled by complex interactions in the tumor microenvironment, where intricate signaling pathways converge to activate a network of specific transcription factors (TF). These TF drive a spectrum of phenotypic changes ranging from a hybrid epithelial/mesenchymal to a pronouncedly mesenchymal phenotype (Hotz et al., 2007; Lamouille et al., 2014; Palamaris et al., 2021; Yang et al., 2023).

Throughout different stages of transdifferentiation, variations in the active efflux of metabolites and drugs mediated by multidrug resistance-associated proteins (MRPs) accompany EMT-induced changes that facilitate cancer progression and drug resistance (Jiang et al., 2017; Santamaria et al., 2019; Duan et al., 2023). MRPs, members of the ATP-binding cassette (ABC) protein superfamily, transport various endogenous substrates, modulating the tumor microenvironment and facilitating drug resistance (El-Awady et al., 2017). Several studies have demonstrated that EMT-related TF control the expression of a complex network of ABC transporters, impacting drug resistance and cell survival (Jiang et al., 2017). High expression of ABC transporters has been reported in less differentiated and more aggressive tumor regions (König et al., 2005; Adamska and Falasca, 2018). Known stromal EMT-inducing signals, such as hypoxia, inflammation, and TGF-beta, enhance the expression of key ABC transporters, including ABCB1 (P-gp), ABCC1 (MRP1), and ABCG2 (BCRP), in various cancers, including PDAC (Jiang et al., 2017; Santamaria et al., 2019; Okada et al., 2021). In PDAC, accumulating evidence shows that the xenobiotic transporter *ABCC4*/MRP4 (multidrug resistance-associated protein 4), plays a pivotal role in cell proliferation, metastasis, and serves as a potential prognostic biomarker and therapeutic target (Zhang et al., 2012; Carozzo et al., 2019; Sahores et al., 2020). Overexpression of MRP4 has been reported in several other cancers, including prostate, ovarian, breast, colorectal, neuroblastoma, hepatocellular, non-small cell lung, and acute myeloid leukemia (Ho et al., 2008; Copsel et al., 2011; Huynh et al., 2012; Zhao et al., 2014; Kochel et al., 2016; Jung et al., 2020; Kryczka et al., 2020; Zhou et al., 2022). Our previous studies have demonstrated that *ABCC4*/MRP4 is highly expressed in poorly differentiated and more malignant PDAC tumors and cell lines, promoting cell proliferation via cAMP extrusion into the extracellular compartment (Carozzo et al., 2019), and contributing to dysregulated transcriptional pathways associated with cell adhesion, chemotaxis, migration, and metastasis (Sahores et al., 2020).

In this study, we aimed to comprehensively investigate the functional implications of altered *ABCC4* expression in the PDAC phenotype, and to elucidate the epigenetic and molecular mechanisms underlying its dysregulated expression. Our findings demonstrate that high *ABCC4* expression promotes EMT programs, in part mediated by changes in TF expression such as pro-epithelial FOXA1 and pro-mesenchymal GATA2, which in turn act at specific *cis*-regulatory elements located at *ABCC4* distal promoter and intron one eliciting a positive feedback loop.

## Materials and methods

### Cell culture

PANC-1, BxPC-3, and HPAF-II human pancreatic cancer cell lines were purchased from the American Type Culture Collection (ATCC, USA) and grown in Dulbecco’s Modified Eagle’s Medium (DMEM) or RPMI-1640 (RPMI) medium (Sigma-Aldrich, USA). The clones with different levels of *ABCC4*/MRP4 expression were previously established and characterized in our laboratory (Carozzo et al., 2019). Medium was always supplemented with 10% FBS (Natocor) and 50 μg/mL gentamicin (Sigma-Aldrich). All cell cultures were maintained at 37°C in a humidified atmosphere with 5% CO_2_. BxPC-3 clones were maintained with hygromycinB (SelleckChem; 200 μg/mL). Cells were authenticated by short tandem repeat profiling (Genetica DNA Laboratories Inc.). All experiments were performed with mycoplasma-free cells and all human cell lines have been authenticated using STR profiling within the last 3 years.

### Animals

Two-month-old male immunodeficient NOD/LtSz-scid/IL-2Rgamma null (NSG; Jackson Labs) mice (27 g±2 g) were bred and maintained under specific pathogen-free conditions in filter-top boxes with a 12 h light/12 h dark cycles at the ININFA animal facility. All studies comply with the ARRIVE guidelines and were carried out in accordance with the UK Animals (Scientific Procedures) Act. The experimental protocols were approved by the corresponding IACUC authorities of the University of Buenos Aires (IBYME; CICUAL N° 014/2017 and N° 016/2021) and all experiments were performed following the Institutional Bio-Safety Committee (IBSC) regulatory framework.

### Xenografts

For subcutaneous tumor xenografts, 2 × 10^6^ BxPC-3 mock or BxPC-3 MRP4+, 5 × 10^6^ PANC-1, or 5 × 10^6^ HPAF-II cells were inoculated (100 µL) into the right inguinal flank of male NSG mice. When tumors reached a maximum diameter of 15mm, mice were euthanized, and xenografts were excised.

### RNA-seq

Total RNA from BxPC-3 mock and MRP4+ cells grown in culture (N = 2) and xenografts (N = 3) was extracted with QuickZol (Kalium Technologies) followed by incubation with 3U of TURBO DNase^®^ (Ambion, Thermo Fisher Scientific Inc.) according to the manufacturer’s instructions. Purified RNA samples (4 µg) were sent to Novogene for library preparation (*TruSeq RNA Sample Preparation Kit v2*, Illumina Inc.) and sequencing (150-nt *paired-end*) on the *Illumina HiSeq2500* platform (Illumina Inc.). Approximately 20 million reads *per* sample were obtained. FASTQ files from BxPC-3 cell cultures and xenografts were processed together with the FASTQ files from PANC-1 scramble and MRP4- clones previously published (Carozzo et al., 2019; Sahores et al., 2020). The following bioinformatics pipeline was used: quality control with FastQC, trimming with fastp, mapping with STAR to the Hg19 human reference genome, and counting with featureCounts. The differentially expressed genes (DEG) were calculated with DESeq2 (padj<0.05, Log2FC > 1). Heatmaps and volcano plots were performed with packages pheatmap and EnhancedVolcanoplot. Functional enrichment was conducted with Enrichr (https://maayanlab.cloud/Enrichr/), selecting the Molecular Signature Database (MSigDB Hallmark, 2020), KEGG (2021 human), WikiPathway (2021 human), and Gene Ontologies (Biological Process, Cellular Component, Molecular Function, 2023) databases, and padj<0.05. Count matrices from PANC-1 and BxPC-3 cultures and xenografts, and full R scripts are available at https://github.com/Gonzalez-Lab/ABCC4.

### Correlation studies of ABCC4/MRP4 levels in PDAC human sample datasets and preclinical models

Pearson correlation analysis was conducted to assess the relationship between *ABCC4* expression levels and marker genes associated with epithelial or mesenchymal programs (Log2) using transcriptomic datasets accessible through public repositories such as GEO (Gene Expression Omnibus) and TCGA (The Cancer Genome Atlas). Additionally, transcriptomic datasets from patient cohorts recruited from various academic hospitals in Europe and the USA were included in the analysis. A total of 13 datasets from different preclinical models and patient samples were analyzed: i) cell lines: Moffit cell lines (GSE71729) (Moffitt et al., 2015), Diaferia cell lines (GSE64558) (Diaferia et al., 2016); ii) primary patient-derived cultured cells (PDC): PDC. Fraunhoffer (Fraunhoffer et al., 2023); iii) patient-derived xenografts (PDX): PDX. Fraunhoffer (Fraunhoffer et al., 2022), PDX. Moffitt (Moffitt et al., 2015); iv) patient primary tumors: TCGA. PAAD (Cancer Genome Atlas Research Network, 2017), Moffit. PDAC (Moffitt et al., 2015), Moffitt. primary (GSE71729) (Moffitt et al., 2015), Gempred (Nicolle et al., 2021), Multi. Stage (Fraunhoffer et al., 2022; 2023), Puleo (Puleo et al., 2018), and Compass (Aung et al., 2018). Pearson correlation coefficients and their statistical significance were calculated using statistical functions in the R programming language. These results were visualized in a heatmap with column hierarchical clustering, employing the R package pheatmap.

### Identification of regulatory clusters on the ABCC4 gene

To examine *cis*-regulatory elements within *ABCC4*, we employed the Integrative Genomics Viewer (IGV) platform to visualize ChIP-seq tracks obtained at the ChIP-Atlas Peak Browser platform (http://chip-atlas.org/peak_browser, Zou et al., 2022), as well as manually curated BED files of epigenetic marks and TF performed in the PDAC cell lines CFAC1 (GSE54560) (Diaferia et al., 2016). The complete *ABCC4* gene, spanning 15 kb upstream of the TSS was visualized. Poised/active *cis*-regulatory elements were identified based on the enrichment peaks corresponding to H3K4me1/H3K27ac marks, *ATAC-seq*, and positive TF binding, such as FOXA1 and GATA2 in PDAC and prostate cancer. The comparative analysis of shared and differential active *cis*-regulatory sites between the PDAC cell lines HPAF-II and PANC-1 was conducted using the Galaxy Community 2022 platform.

### Chromatin immunoprecipitation (ChIP) assays

HPAF-II and PANC-1 tumor xenografts minced tissue was cross-linked in 1% formaldehyde/PBS for 15 min. Dynabeads (Life Technologies) were blocked with BSA (Sigma-Aldrich) and incubated with anti-FOXA1 (5µg, sc-514695 Santa Cruz Biotechnology), anti-GATA2 (5µg, sc-267 Santa Cruz Biotechnology), or normal rabbit IgG (5µg, 12–370 Millipore) antibodies. Chromatin shearing was carried out using a temperature-controlled cold-water bath and rotating sonicator (Bioruptor Pico, Diagenode). Immunoprecipitation was conducted overnight at 4°C with equal amounts of chromatin lysate (25–30 μg) per sample. DNA-protein complexes were then disassociated at 65°C with proteinase K (Life Technologies) for 2 h after RNaseA treatment. DNA extraction was performed using phenol/chloroform extraction and suspended in 10 mM Tris buffer. PCR amplification of ChIP-derived DNA was carried out using the ABIPrism7500 sequence detection system (Applied Biosystems). Enrichment of FOXA1 and GATA2 was determined using specific ChIP primers designed to amplify the selected clusters at the distal promoter (DP) and intron 1 (clusters 1-3) (sequences in Supplementary Table S1). Each sample was analyzed in duplicate using 4 pmol of each primer, 1X HOT FIREPol’ EvaGreen qPCR Mix Plus (Solis Biodyne), and 5 μL of the immunoprecipitated (IP) and input DNA samples (1 ng/μL) in a final reaction volume of 13 μL. Data was quantified as the % IP over input.

### Statistical analysis

Data are presented as mean ± SEM. Statistical analyses were performed using two-way ANOVAs, followed by Tukey *post-hoc* test, to compare two factors (group and growth setting) and their interaction. For data that did not meet parametric test assumptions, Kruskal-Wallis ANOVA on ranks was applied, followed by paired comparisons. Statistical analyses were conducted using the software InfoStat 2010 and R packages. Results were considered statistically significant at p<0.05.

## Results

### Effect of the modulation of ABCC4/MRP4 levels on the transcriptome of PDAC cell lines

**FIGURE 1:**
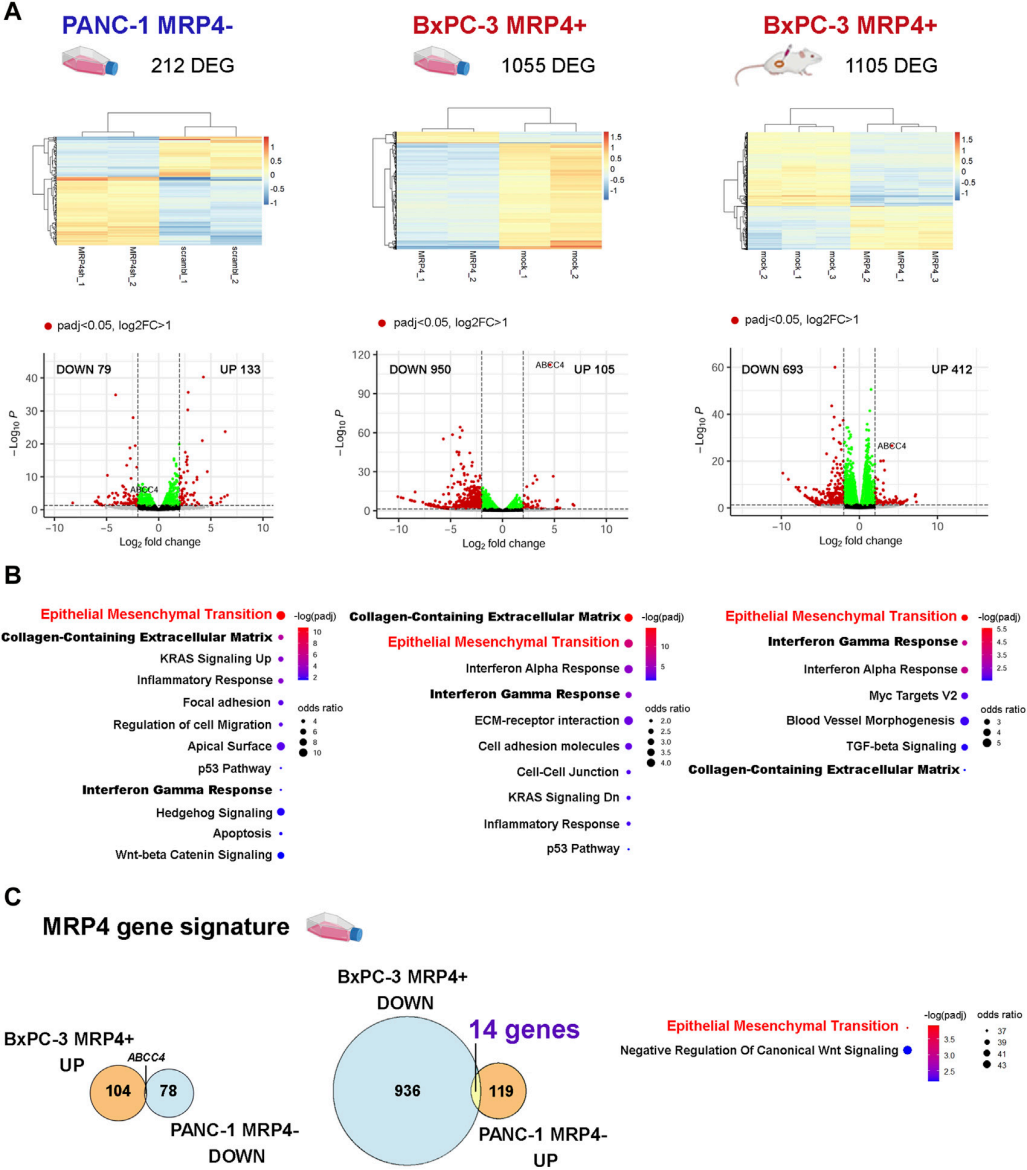
Effect of the modulation of *ABCC4*/MRP4 levels on the transcriptome of PDAC cells. We performed *RNA-seq* analysis on PANC-1 cells with silenced *ABCC4* expression (left: PANC-1 MRP4- vs. scramble) and BxPC-3 cells with *ABCC4*/MRP4 overexpression (BxPC-3 MRP4+ vs. mock). The transcriptome of BxPC-3 cells was analyzed in both cultured cells (middle) and xenografts derived from sc. Inoculation in NGS mice (right). FASTQ files were aligned to the human reference genome (hg19) using STAR-featureCounts, and count matrices were analyzed with DESeq2. **(A)** Unsupervised clustering of the differentially expressed genes (DEG) identified in PANC-1 and BxPC-3 samples (z-score). **(B)** Volcano plots illustrating downregulated (DOWN) and upregulated (UP) DEG in PANC-1 and BxPC-3 samples. **(C)** Functional enrichment analysis performed using the Enrichr platform. Bubble charts depict the significance level (blue to red) and odds ratio (bubble size) for the most significant terms obtained for PANC-1 and BxPC-3 sample DEG (padj<0.05). Bold and red lettering highlights the most significant shared term across all experimental models. **(D)** Left: Venn diagrams illustrate the “MRP4 gene signature” derived from the analysis of DEG showing opposite changes between PANC-1 MRP4- and BxPC-3 MRP4+ cell cultures (padj<0.05). Right: Functional enrichment analysis of the “MRP4 gene signature.”

To further elucidate the effects of *ABCC4*/MRP4 on the PDAC phenotype, we performed transcriptomic analysis comparing PANC-1 cells with reduced *ABCC4* expression (MRP4-) to control (scramble) cells, and BxPC-3 cells with enhanced *ABCC4*/MRP4 levels (MRP4+) to control (mock) cells, growing in culture and in subcutaneous xenografts. Figure 1 illustrates the differential gene expression analysis (DESeq2) conducted on PANC-1 scramble vs. MRP4- and BxPC-3 mock vs. MRP4+ cell cultures and xenografts (padj<0.05; Log2FC > 1). Consistent with expectations, PANC-1 MRP4- cell cultures exhibited decreased *ABCC4* levels, while BxPC-3 MRP4+ cell cultures and xenografts showed increased *ABCC4* levels vs. control samples (Figure 1A). Functional enrichment analysis showed that altered expression of *ABCC4*/MRP4 was most significantly associated with the *Epithelial Mesenchymal Transition*, *Collagen-Containing Extracellular Matrix*, and *Interferon Gamma Response* signatures (Figure 1B; Supplementary Table S2). We further established a “MRP4 gene signature” by identifying genes that exhibited opposite changes in expression between PANC-1 MRP4- and BxPC-3 MRP4+ cell culture settings. We identified 14 genes significantly upregulated in PANC-1 MRP4- and downregulated in BxPC-3 MRP4+ samples (Figure 1C; Supplementary Table S3). Functional enrichment analysis revealed a significant association with the *Epithelial Mesenchymal Transition* term, underscoring the strong correlation between *ABCC4*/MRP4 modulation and alteration in the EMT process in PDAC cells.

### ABCC4/MRP4 expression is associated with the EMT program in PDAC

**FIGURE 2:**
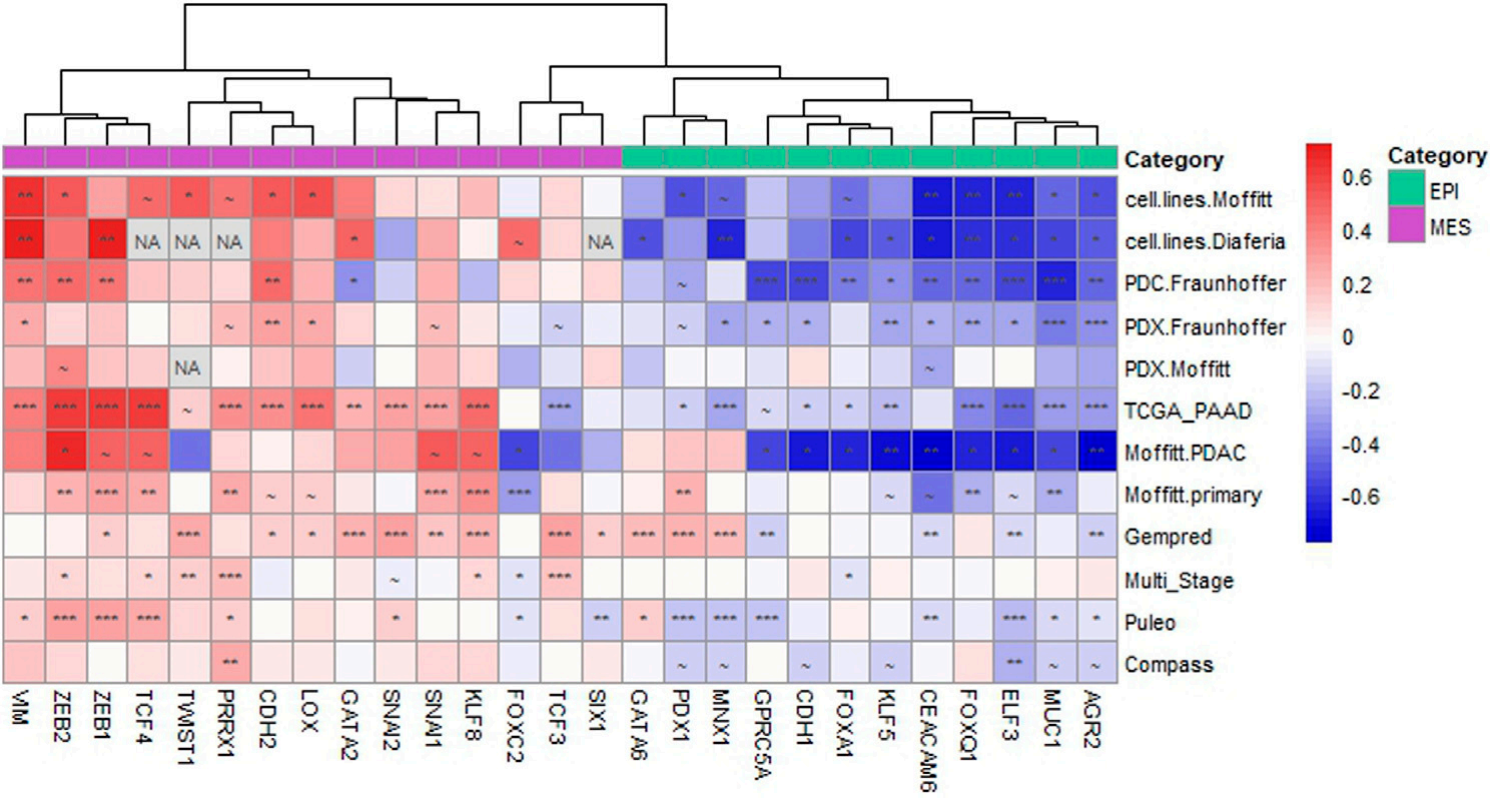
*ABCC4* correlation with epithelial and mesenchymal markers in PDAC. Hierarchical clustering of the Pearson’s correlation coefficient (R) calculated between the Log2-transformed expression levels of ABCC4 and the selected epithelial (EPI) and mesenchymal (MES) genes across various PDAC models. PDAC models include cell lines (GSE71729 from (Moffitt et al., 2015); GSE64558 from (Diaferia et al., 2016)), primary patient-derived cultured cells (PDC; Fraunhoffer et al., 2023), and patient-derived xenografts (PDX, Moffitt et al., 2015; Fraunhoffer et al., 2022). PDAC primary tumor samples encompass datasets such as TCGA_PAAD (Cancer Genome Atlas Research Network, 2017), Moffitt. PDAC and Moffitt. primary (GSE71729 from Moffitt et al., 2015), Gempred (Nicolle et al., 2021), Multi_Stage (Fraunhoffer et al., 2022; Fraunhoffer et al., 2023), Puleo (Puleo et al., 2018), and Compass (Aung et al., 2018). The color scale indicates negative (blue) to positive (red) correlations. Symbols denote significance levels: ∼: p< 0.1, *: p< 0.05, **: p< 0.01, ***: p< 0.005. “NA” indicates gene expression not reported in the dataset.

To comprehensively evaluate the association between *ABCC4*/MRP4 and the EMT process, we analyzed transcriptomic data from PDAC primary tumors (Moffitt et al., 2015; Research Network, 2017; Aung et al., 2018; Puleo et al., 2018; Nicolle et al., 2021; Fraunhoffer et al., 2022; Fraunhoffer et al., 2023; Cancer Genome Atlas), as well as preclinical PDAC models including cell lines, primary patient-derived cells (PDC), and patient-derived xenografts (PDX) (Moffitt et al., 2015; Diaferia et al., 2016; Fraunhoffer et al., 2022; Fraunhoffer et al., 2023). We evaluated the correlation between *ABCC4* levels and the expression of well-established markers and TF involved in the epithelial (EPI) and mesenchymal (MES) programs (Moffitt et al., 2015; Diaferia et al., 2016; Porter et al., 2019). Hierarchical clustering of the Pearson’s correlation coefficient (R) revealed a negative correlation between *ABCC4* levels and the EPI markers, and a positive correlation with the MES markers (Figure 2). Interestingly, the EPI markers clustered together, while the MES markers exhibited the strongest positive correlation in key genes such as VIM and ZEB1/2. This correlation trend was consistent across PDAC samples and experimental models lacking the stromal compartment, including cell lines, PDC and PDX, suggesting that the observed correlations specifically reflect gene expression within the tumor parenchyma. Additionally, we assessed the expression levels of selected EPI and MES markers in our *RNA-seq* experiments. We found that upregulation of *ABCC4*/MRP4 was associated with the downregulation of several EPI markers and the dysregulation of several MES markers, particularly evident in the xenograft model (Supplementary Table S4).

**FIGURE 3:**
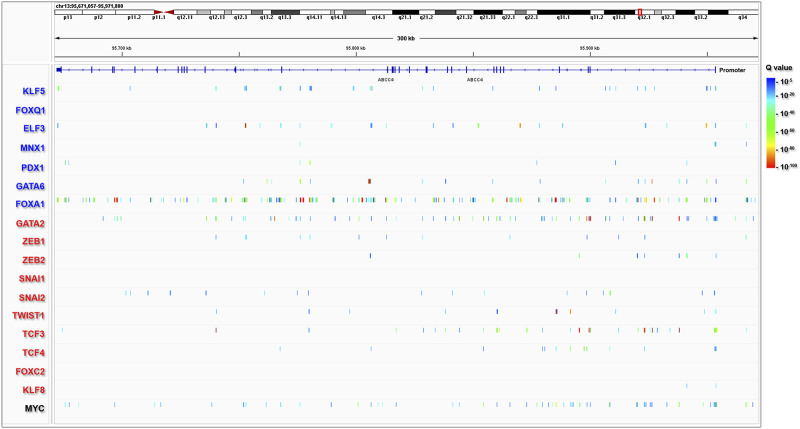
Epithelial and mesenchymal transcription factors binding sites at *ABCC4* gene. *ChIP-seq* peaks representing epithelial TF (blue lettering) and mesenchymal TF (red lettering). The data were obtained from the ChIP-Atlas portal using the Peak Browser (selected settings: *H. Sapiens* Hg19, all cells, threshold Q value < 10^−5^), and the visualization was performed using Integrative Genome Browser (IGV) software. The colors displayed in the IGV tracks indicate the statistical significance (Q value) of the peak called with MACS2.

To explore whether *ABCC4* may be directly regulated by the EPI vs. MES transcriptional programs, we curated available data on reported *ChIP-seq* binding sites for EPI and MES TF. Figure 3 shows the promoter and entire gene body of *ABCC4* on the negative strand of chromosome 13, along with the peaks called for EPI TF (blue lettering) and MES TF (red lettering) across various normal and neoplastic cell types. Among the selected TF, certain ones exhibited high coverage, such as FOXA1 and GATA2. Additionally, we observed significant coverage of MYC, a master regulator of cell proliferation known to directly control *ABCC4* transcription (Huynh et al., 2012; Jung et al., 2020). These results indicate that *ABCC4* expression may be influenced by the interplay between EPI and MES transcriptional programs, with its upregulation being associated with EMT and cell proliferation.

### Dissecting ABCC4 cis-regulatory elements and FOXA1/GATA2 binding in PDAC

**FIGURE 4:**
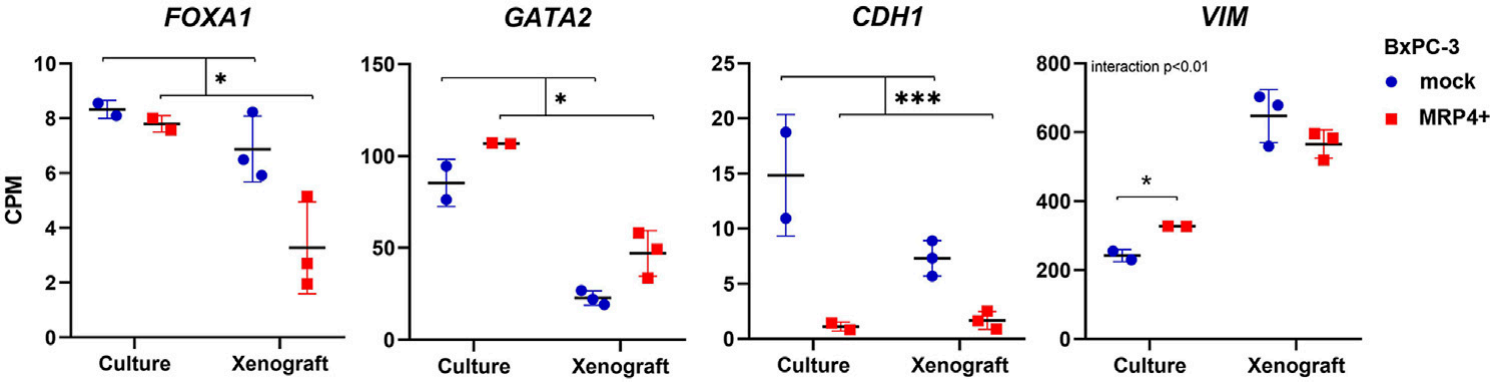
EMT transcription factors and markers expression in BxPC-3 cells with *ABCC4*/MRP4 overexpression. Transcript levels in BxPC-3 MRP4+ vs. mock cultures and xenografts obtained in the RNA-seq experiments are shown in counts per million (CPM). Data are presented as mean ± SEM (N = 2-3). Two-way ANOVA, followed by Tukey post test when interaction p< 0.05. *p< 0.05, ***p< 0.001.

We further focused on elucidating the role of FOXA1 and GATA2, two pioneer TF known to interact with *ABCC4* elements and regulate gene expression (Wu et al., 2014; Rodriguez-Bravo et al., 2017). FOXA1 is recognized as pro-epithelial TF in PDAC, contributing to tumorigenesis and metastasis by mechanisms that involve *cis*trome reprogramming toward aberrant embryonic endoderm developmental programs (Roe et al., 2017). Conversely, GATA2 is significantly upregulated in more undifferentiated and high-grade PDAC cell lines and xenografts (Diaferia et al., 2016). Actually, in our *RNA-seq* studies we found that *ABCC4*/MRP4 overexpression in BxPC-3 induces a shift in these TF levels, decreasing *FOXA1* and increasing *GATA2*, consistent with an EPI-to-MES switch in structural markers such as *CDH1* and *VIM* (Figure 4; Supplementary Table S4).

**FIGURE 5:**
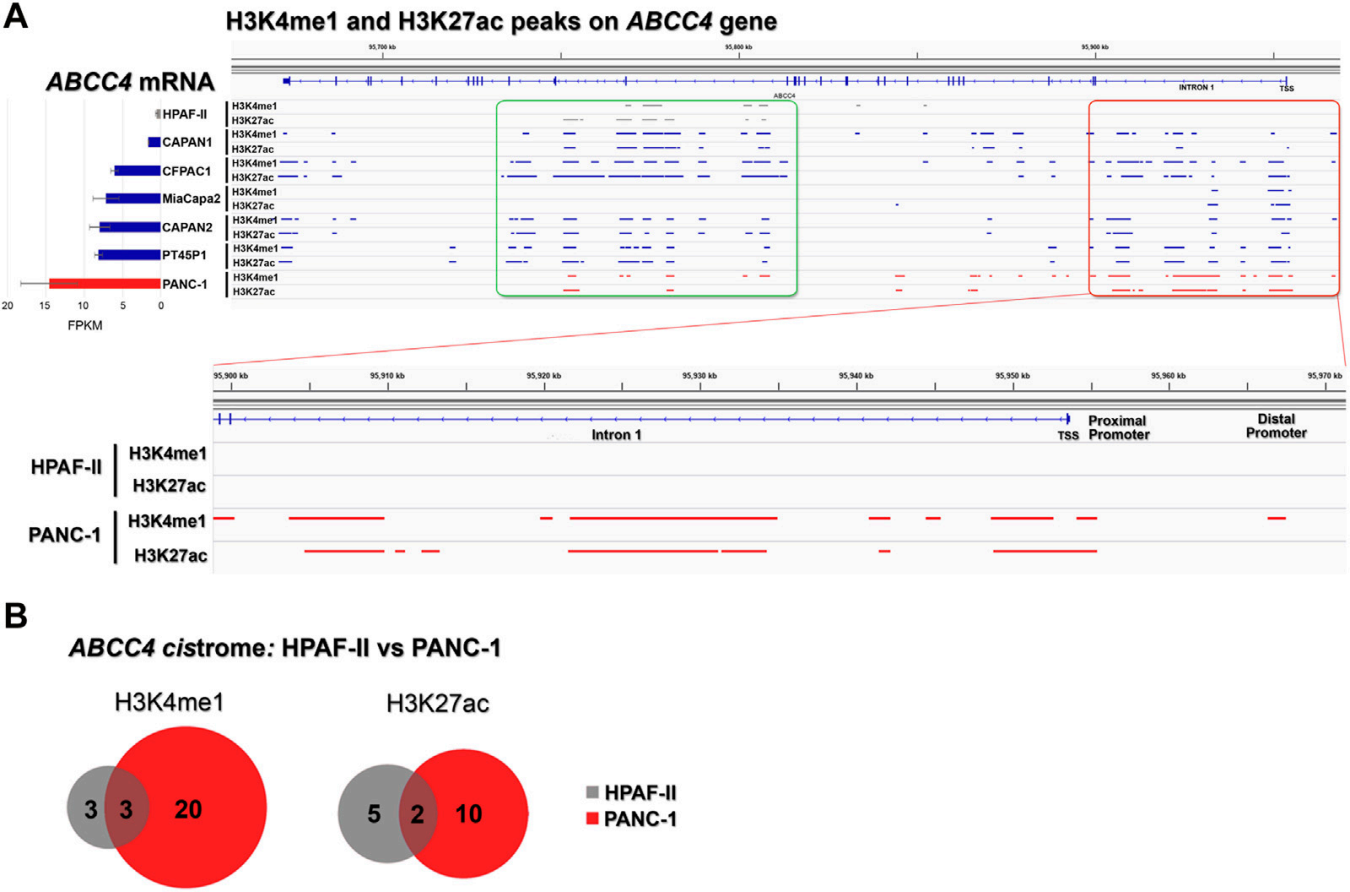
*Cis-regulatory elements within ABCC4 in PDAC cell lines.* **(A)** *ABCC4 transcri*pt levels (left) and the genome-wide distribution of poised/active *cis*-regulatory elements H3K4me1/H3K27ac (right) in seven PDAC cell lines: low-grade HPAF-II, CAPAN1, CAPAN2, and CFPAC1, and high-grade MiaPaCa2, PT45P1, and PANC-1. Data were retrieved from GSE64560 (*RNA-seq*) and GSE64557 (*ChIP-seq*) datasets (Diaferia et al., 2016), and tracks were visualized using Integrative Genome Browser (IGV) software. **(B)** Intersection analysis for H3K4me1/H3K27ac peaks in HPAF-II vs. PANC-1 cell lines, illustrating shared and exclusive active sites across the *ABCC4* gene. Analysis was performed using Galaxy/Cistrome tools (http://cistrome.org/ap/).

To delve into the mechanisms regulating *ABCC4* expression, we queried epigenomic and transcriptomic data from PDAC cell lines exhibiting differential endogenous levels of MRP4. First, we defined the active TF clusters along the *ABCC4* gene, typically neighbored by the *cis*-regulatory element epigenetic signature, composed of histones marks H3K4me1 (indicative of poised sites) and H3K27ac (indicative of fully active sites) (Calo and Wysocka, 2013), in seven low-grade (HPAF-II, CAPAN1, CAPAN2, and CFPAC1), and high-grade (MiaPaCa2, PT45P1, and PANC-1) PDAC cell lines, sorted by increasing *ABCC4* expression levels (*RNA-seq*, left), alongside the peaks for H3K4me1/H3K27ac marks on the *ABCC4* gene (*ChIP-seq*, right). Notably, two large regulatory regions (green and red rectangles) were delineated, displaying higher density of both marks across all cell lines. In particular, the red-marked region spans from 18 kb upstream of the transcription start site (TSS) to the second exon, encompassing both distal and proximal promoters. Substantial disparities in H3K4me1/H3K27ac content were observed within this region between cell lines with varying *ABCC4* expression levels. This contrast is particularly evident between HPAF-II (gray tracks) and PANC-1 (red tracks) cell lines, representing opposite ends of *ABCC4* expression. While no peaks were detected for either mark in HPAF-II cells, PANC-1 cells exhibited the highest coverage among the 7 cell lines analyzed. Intersection analysis of positive H3K4me1 and H3K27ac sites throughout the entire *ABCC4* gene, revealed that HPAF-II cells displayed fewer posed/active regulatory sites, whether exclusive or shared, while PANC-1 cells exhibited a greater number of differentially marked sites (Figure 5B). This indicates heightened regulatory activity on the *ABCC4* gene in the cell line with elevated expression levels.

**FIGURE 6:**
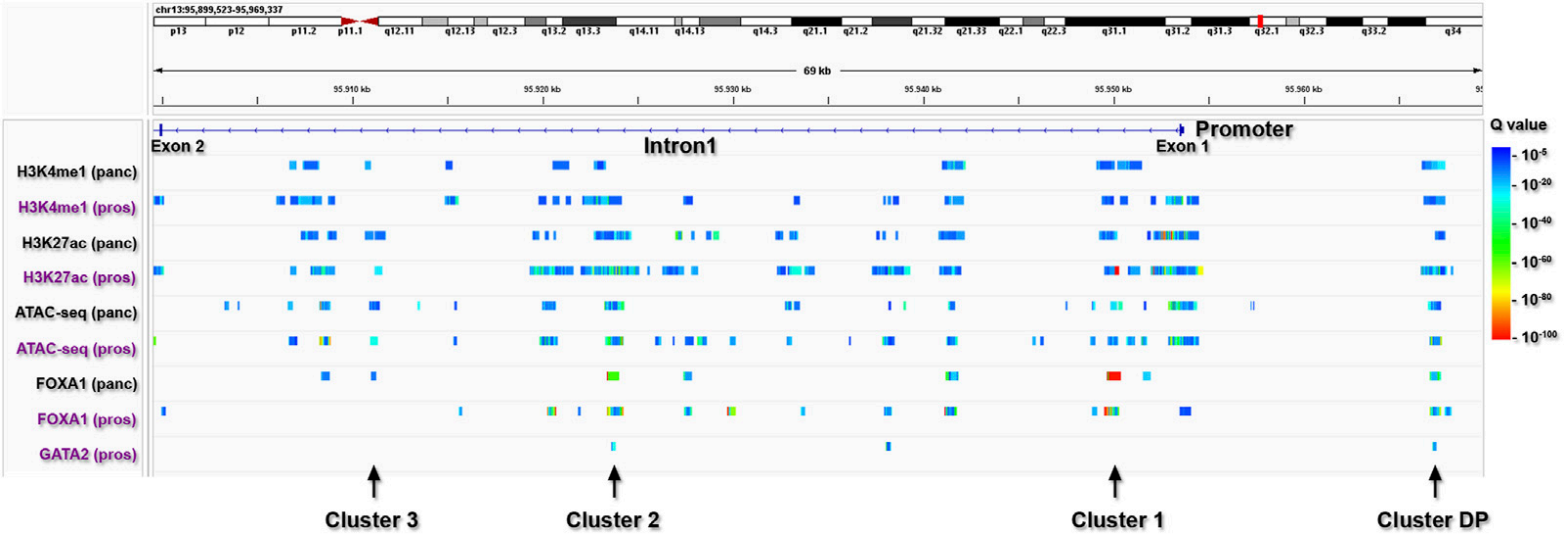
Identification of primary regulatory clusters within *ABCC4*. *ChIP-seq* peaks from pancreatic (panc: black lettering) and prostate (pros: purple lettering) cells, indicating poised/active *cis*-regulatory elements marked by H3K4me1/H3K27ac and *ATAC-seq* peaks, as well as FOXA1 and GATA2 binding sites within *ABCC4*.

To further delineate the primary regulatory regions within the *ABCC4* gene in PDAC, we specifically focused on identifying *cis*-regulatory elements within *ABCC4* (Figure 6). For this study, we selected tracks available for H3K4me1/H3K27ac, *ATAC-seq*, and FOXA1 on pancreatic and prostate cell types, which include several PDAC cell lines like CFPAC1, PT45-P1, BxPC-3, and PANC-1, and prostate cancer cell lines like LnCAP, where the control of *ABCC4* transcription by FOXA1/GATA2 binding at intron one sites was first documented (Wu et al., 2014). Notably, we found a highly similar *cis*trome and FOXA1 binding between pancreatic and prostate cancer cell lines tracks (Figure 6). We also evaluated the genomic distribution of H3K4me1/H3K27ac, FOXA1, and other TF involved in pancreatic tumor development on the CFPAC1 PDAC cell line (GSE54560) (Diaferia et al., 2016) (Supplementary Figure S1). It is noteworthy that CFPAC1 cells exhibit intermediate *ABCC4* levels and an epigenetic profile within intron one similar to that observed in PANC-1 cells (Figure 5; Supplementary Figure S1). This analysis enabled us to define four clusters of interest within *ABCC4*: a distal promoter (DP) site, situated at −13 kb, along with three clusters located within intron one at positions 3.5 (cluster 1), 29.9 (cluster 2), and 42.5 (cluster 3) kb (Figure 6). These regulatory regions were chosen based on the presence of combined FOXA1/GATA2 binding (clusters DP and 2) or exclusive FOXA1 binding (clusters one and 3).

**FIGURE 7:**
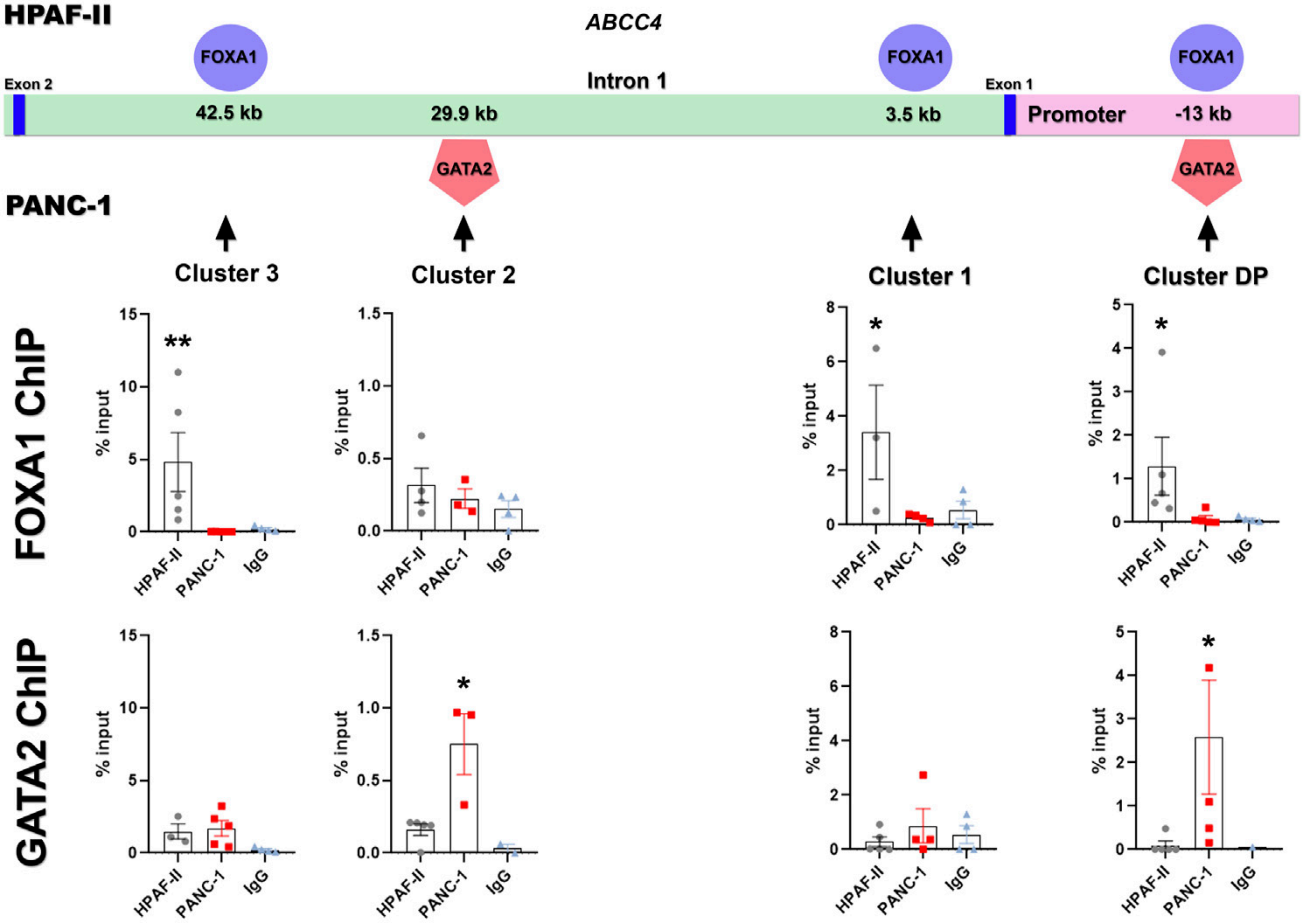
Identification of FOXA1/GATA2 binding within *ABCC4* in PDAC cell lines. Specific binding of FOXA1 and GATA2 at the identified regulatory clusters within the *ABCC4* gene in HPAF-II and PANC-1 xenografts, determined by *ChIP-qPCR*. Data are presented as mean ± SEM. Kruskal-Wallis test (N = 3-5); *p< 0.05 vs. negative control with IgG antibody.

Next, we aimed to characterize FOXA1 and GATA2 effective binding to the identified regulatory clusters in *in-vivo* PDAC models. For this purpose, we generated HPAF-II and PANC-1 xenografts and performed *ChIP-qPCR* analysis to examine the specific binding of FOXA1 and GATA2 at the selected regulatory clusters within the *ABCC4* gene. As expected, HPAF-II xenografts showed glandular structures, typical of highly differentiated tumors, with higher FOXA1 expression, whereas PANC-1 xenografts exhibited solid and compact tumor sheets without lumens, indicative of poor differentiation, along with higher *GATA2* and *ABCC4* expression (Supplementary Figure S2). Consistently, we found significant FOXA1 enrichment at clusters DP, 1, and three in HPAF-II compared to PANC-1 xenografts (p< 0.05; Figure 7). Conversely, PANC-1 tumors displayed notable GATA2 enrichment at clusters DP and 2, relative to HPAF-II (p< 0.05, Figure 7).

**FIGURE 8:**
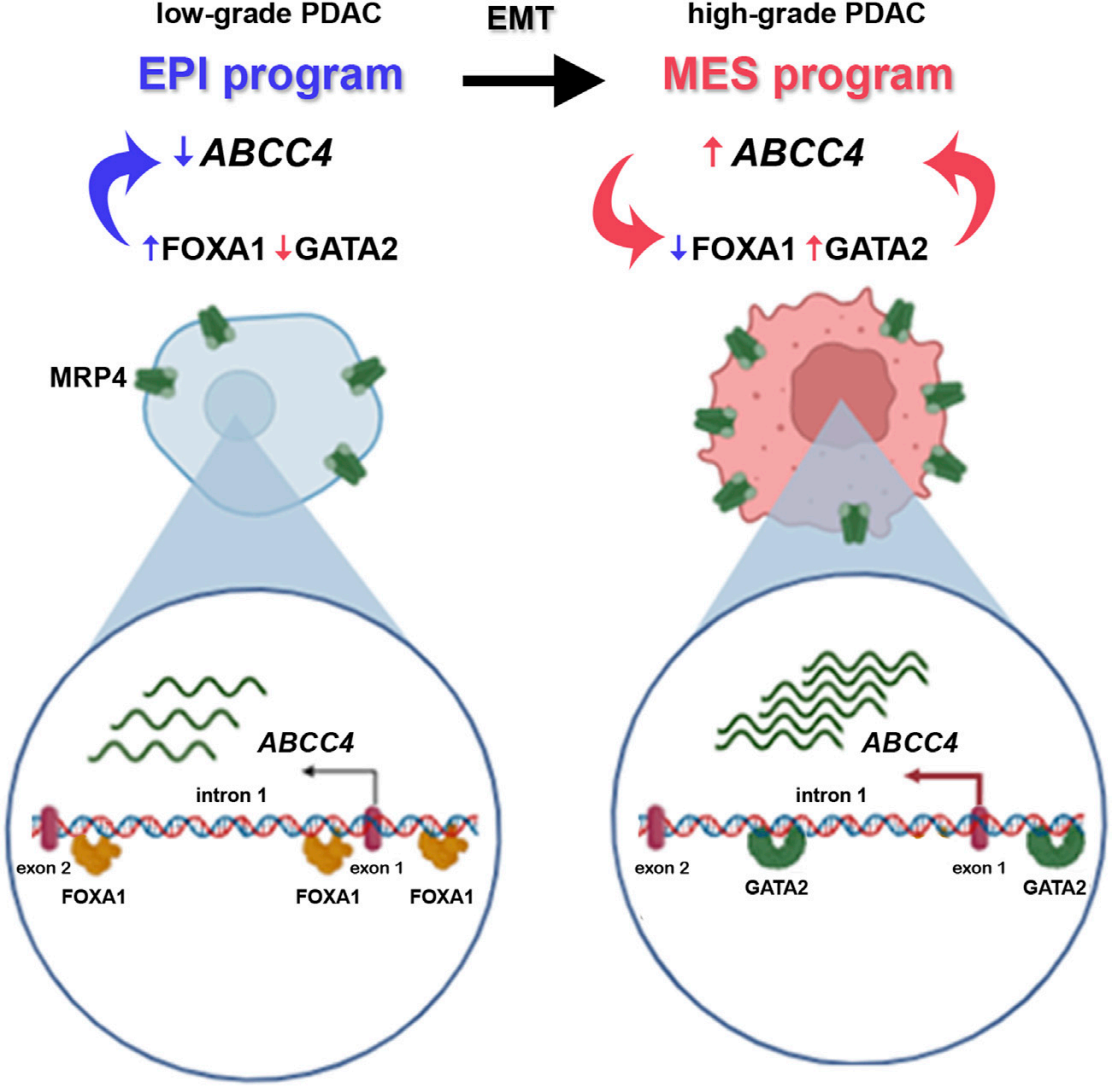
Schematic illustration of the epithelial-to-mesenchymal transition (EMT) program in PDAC progression, from low-to high-grade tumors. Low-grade tumors exhibit high expression of epithelial (EPI) program TF (in blue), like FOXA1. FOXA1 binds to the promoter of the *ABCC4* gene and ensures low levels of the protein it encodes, MRP4. During EMT, EPI gene expression decreases as mesenchymal (MES) program TF, such as GATA2, are upregulated (in pink). This TF also binds to *cis*-regulatory elements within the *ABCC4* gene, triggering its transcription and heightening *ABCC4/*MRP4 levels in high-grade tumors. In turn, increased *ABCC4* expression further diminishes FOXA1 and boosts GATA2 levels ensuring the heightened expression of the transporter through a positive feedback loop.

The results presented here can be summarized in Figure 8: hightened *ABCC4* expression activates EMT programs, downregulating EPI TF expression like FOXA1, while upregulating MES TF, such as GATA2. This switch in TFs levels then operates on distinct *cis*-regulatory elements situated within the *ABCC4* distal promoter and intron 1, triggering a positive feedback loop. Targeting these dynamic transitions and programs could be a promising strategy for PDAC treatment.

## Discussion

In recent years, accumulating evidence has highlighted *ABCC4*/MRP4 as a promising prognostic marker in various cancers, including PDAC. The results presented herein further delve into the functional implications of elevated *ABCC4* levels in PDAC by establishing its association with the EMT, a pivotal process in cancer progression, aggressiveness, and resistance to chemotherapy (Palamaris et al., 2021). Our study reveals a positive correlation between *ABCC4* expression and a pro-mesenchymal phenotype, along with a negative correlation with pro-epithelial TF and markers across multiple transcriptomic datasets comprising PDAC tumor samples and preclinical models. Moreover, modulation of *ABCC4* levels in PANC-1 and BxPC-3 cells resulted in dysregulation of genes predominantly associated with the EMT signature. Overexpression of *ABCC4*/MRP4 led to increased pro-mesenchymal *GATA2* and decreased pro-epithelial *FOXA1* and *CDH1* expression. Additionally, we established a “MRP4 gene signature” in PDAC by identifying genes exhibiting opposite expression patterns in MRP4- vs. MRP4+ cell cultures, which are significantly associated with the EMT signature. Notably, *ABCC4* expression has also been linked to the EMT process in colorectal cancer, as evidenced by its correlation with EMT markers in primary tumors datasets, and the induction of a pro-mesenchymal phenotype and increased *ABCC4* expression upon overexpression of SNAI2 in HT29 colorectal adenocarcinoma cells (Kryczka et al., 2020).

In this study, we build upon previous findings and compared the transcriptomic profiles of PANC-1 cells with decreased *ABCC4*/MRP4 levels and BxPC-3 cells with increased *ABCC4*/MRP4 levels, both in cell culture and xenograft models. Functional enrichment studies of the DEG showed common pathways across different experimental conditions, such as the EMT, the collagen-containing extracellular matrix, and the interferon gamma response signatures. Collagen fibrils constitute a major component of the desmoplastic stroma characteristic of PDAC, facilitating tumor progression and metastasis by creating a stiff and hypoxic microenvironment that exerts selective pressure on neoplastic cells (Zhao et al., 2022). The collagen signature may reflect the acquisition of a pro-mesenchymal phenotype by cells undergoing EMT, as fibroblasts from the mesenchymal compartment are the primary contributors to collagen secretion and the fibrotic components of the extracellular matrix (Zhao et al., 2022). Moreover, in both MRP4- and MRP4+ cell culture settings, we detected key signatures related to PDAC pathogenesis, specifically involving the KRAS and p53 pathways, as well as the inflammatory response, that play crucial roles in pancreatic tumor development and progression (Hausmann et al., 2014; Zhao et al., 2022). Moreover, these pathways also enhance the expression of key ABC transporters (Jiang et al., 2017; Santamaria et al., 2019; Okada et al., 2021).

The pioneer TF FOXA1 and GATA2 have been previously linked to the regulation of *ABCC4* expression in prostate cancer (Wu et al., 2014), where MRP4 has been validated as a clinically relevant prognostic marker associated with metastasis (Ho et al., 2008; Montani et al., 2013). In LnCAP prostate cancer cells, FOXA1 and GATA2 independently bind to the *ABCC4* gene and recruit chromatin loop-forming factors such as MED1 from distal sites to the cluster two region, allowing androgen receptors to bind and promote transcription upon hormone stimulation (Wu et al., 2014). Importantly, GATA2 overexpression in prostate tumor has been associated with increased motility and invasiveness, proliferation, tumorigenicity, and chemoresistance (Rodriguez-Bravo et al., 2017), whereas in PDAC it was found increased in high-grade tumors and cell lines (Diaferia et al., 2016). Herein, we describe four sites for *ABCC4* regulation that are differentially accessed by FOXA1 in low-grade HPAF-II or by GATA2 in high-grade PANC-1 tumors. This is consistent with the regulatory mechanism reported in prostate cancer. Our results support a model of endogenous *ABCC4* regulation, where pro-epithelial programs characteristic of low-grade PDAC maintain basal levels of transporter expression, and as pro-mesenchymal programs are activated, the specific binding of TF such as GATA2 triggers *ABCC4* transcription, leading to elevated transporter levels and EMT progression.

Augmented expression of ABC transporters has been associated with aggressive, invasive, and chemoresistant cancers (Fletcher et al., 2010; Muriithi et al., 2020; Duvivier et al., 2024), and EMT TF have multiple binding sites at ABC transporters genes (Saxena et al., 2011). Overexpression of certain MES TF, such as TWIST, SNAI1, and FOXC2, has been linked to increased transporters expression and chemoresistance, while their depletion enhances drug sensitivity (Zhuo et al., 2008; Hoshino et al., 2009; Kurrey et al., 2009; Saxena et al., 2011). Our findings reveal that several EPI and MES TF have binding sites within *ABCC4*. Noteworthy, FOXA1 and GATA2 exhibited a high density of binding sites along with MYC, a pro-oncogenic regulator of cell proliferation (Sodir and Evan, 2011) that has been demonstrated to control *ABCC4* transcription (Huynh et al., 2012; Jung et al., 2020). Here, we show that *ABCC4*/MRP4 overexpression in BxPC-3 increased *GATA2*, which in turn can bind *ABCC4 cis*-regulatory elements at the distal promoter and intron one to promote its transcription, creating a positive feedback that could sustain EMT progression. As GATA2 has been proven to be undruggable due to its structure (Kumar et al., 2012; Rodriguez-Bravo et al., 2017), targeting *ABCC4*/MRP4 could serve to control GATA2 upstream and downstream pathways.

Altogether, the results presented in this study broaden our understanding of the functional roles of *ABCC4*/MRP4 in PDAC pathophysiology and associate this transporter to the basal-like/squamous PDAC phenotype, that exhibit worse prognosis and poor response to chemotherapy (Espinet et al., 2022), thus the overall necessity for effective options to treat these patients. Our results also enable the identification of specific *cis*-regulatory elements and TF that control *ABCC4* expression, potentially serving as therapeutic targets on their own, thus opening avenues for gene therapy interventions. In conclusion, this work contributes to the understanding of *ABCC4*/MRP4’s role in inducing treatment resistance, independent of its function in extruding chemotherapeutic drugs, but rather as a driver of EMT. This likely occurs through modulating levels of endogenous metabolites and signaling molecules within the tumor microenvironment. Previous research has indicated that genetic ablation of *ABCC4* has minimal effects on healthy tissues, supporting the potential of inhibiting *ABCC4*/MRP4 as a viable therapeutic strategy, especially in cases of upregulation in diseased tissues (Russell et al., 2008; Yaneff et al., 2019). Additionally, our findings demonstrate a functional connection between *ABCC4*/MRP4 levels and collagen content, inflammation, and communication pathways like Wnt signaling that are crucial players in the tumor-stroma interface (Ram Makena et al., 2019). In these lines, our future investigations will focus on elucidating the impact of MRP4 pharmacological inhibition on EMT and tumor progression, and the cross-communication with the stromal compartment, in *in vivo* PDAC models. In summary, the results presented herein further reinforce the significance of *ABCC4*/MRP4 as a valuable prognostic marker and a potential therapeutic target in PDAC, underscoring the need for additional studies in this area.

## Data Availability

The datasets presented in this study can be found in online repositories. The names of the repository/repositories and accession number(s) can be found below: https://www.ncbi.nlm.nih.gov/geo/, GSE71729. https://www.ncbi.nlm.nih.gov/geo/, GSE64558. https://www.cancer.gov/ccg/research/genome-sequencing/tcga/studied-cancers/pancreatic-ductal-adenocarcinoma-study, TCGA-PAAD. https://www.ebi.ac.uk/arrayexpress/, E-MTAB-6134. https://www.ebi.ac.uk/arrayexpress/, E-MTAB-5039. https://www.ebi.ac.uk/ena, EGAS00001002543. Count matrices from PANC-1 and BxPC-3 cultures and xenografts, and full R scripts are available at https://github.com/Gonzalez-Lab/ABCC4. PDC.Fraunhoffer, Gempred and Multi stage cohorts’ datasets are available from the corresponding author on reasonable request.
