# Supplementary material for: The xenobiotic transporter ABCC4/MRP4 promotes epithelial mesenchymal transition in pancreatic cancer
Source: Front Pharmacol. 2024 Jul 24;15:1432851. doi: 10.3389/fphar.2024.1432851 (PMC11303182; doi:10.3389/fphar.2024.1432851)
Supplement: Supplementary file 1 [file DataSheet1.zip › Data Sheet 1.PDF]

Supplementary information

Table S1. Primer sequences used to evaluate FOXA1 and GATA2 enrichment at *ABCC4* clusters.

Cluster	Location (- strand)	Forward	Reverse	Product (bp)	T
PD	chr13:95966638- 95967187	CGTGGTGCTAGATTACATCA	ATAAAGCCCAGAGAAAGGTG	193	60
Cluster 1	chr13:95949488-95950387	TTTTGTGCCTTAGCCTCCTG	GCTGCCAGTTCACAAGTTCA	191	60
Cluster 2	chr13:95923238-95924037	TTACAGCCCTGTCTCTGACT	CAAGACGACCCAAGAAATAG	168	60
Cluster 3	chr13:95910488-95911437	CGGGAATGTGGGAGACTGTC	GAAACTCATGGTGGGGTGCT	130	60

Table S2. Functional enrichment analysis

Excel file

Table S3. MRP4 signature genes

Excel file

Table S4. Effect of *ABCC4* modulation on epithelial and mesenchymal markers expression. Statistical analysis (DESeq2) obtained for epithelial (EPI) and mesenchymal (MES) genes in PANC-1 MRP4- and BxPC-3 MRP4+ cultures and xenografts. Downregulated genes appear in blue (Log2FC<0; padj<0.05),and upregulated genes in red (Log2FC>0; padj<0.05), NA: not enough expression for analysis.

		**PANC-1 MRP4-**		**BxPC-3 MRP4+**			
		Cell Cultures		Cell Cultures		Xenografts	
Category	Gene	Log2FC	padj	Log2FC	padj	Log2FC	padj
EPI	CDH1	NA	NA	-3.619	9.66E-15	-2.011	2.89E-05
	MUC1	0.638	0.449	-1.104	0.584	-3.434	2.96E-05
	CEACAM6	NA	NA	NA	NA	NA	NA
	AGR2	0.378	0.894	NA	NA	NA	NA
	GPRC5A	-0.671	0.396	-0.163	0.933	-1.198	0.002

	FOXA1	NA	NA	-0.010	0.995	**-0.985**	**0.045**
	KLF5	NA	NA	0.307	0.654	-0.698	0.014
	FOXQ1	-0.856	0.041	0.416	0.844	-1.080	0.355
	ELF3	NA	NA	NA	NA	NA	NA
	PDX1	NA	NA	-0.973	0.048	-0.268	0.725
	MNX1	-0.078	0.999	0.114	0.922	0.440	0.081
	GATA6	-0.537	0.999	0.961	0.001	0.141	0.755
MES	VIM	0.048	0.999	0.510	0.078	-0.092	0.604
	CDH2	1.515	0.702	0.762	0.003	0.000	0.999
	LOX	1.012	0.892	-0.923	0.075	-1.305	1.40E-08
	GATA2	-0.528	0.631	0.414	0.208	**1.147**	**5.05E-08**
	SNAI1	-0.787	0.573	-0.692	0.521	0.325	0.791
	SNAI2	NA	NA	-0.867	0.002	-0.128	0.513
	ZEB1	0.041	0.999	-0.068	0.962	-0.754	0.007
	ZEB2	NA	NA	0.440	0.373	-0.553	0.103
	TCF3	-0.551	0.141	0.040	0.967	0.554	3.21E-06
	KLF8	NA	NA	NA	NA	4.449	0.012
	TWIST1	NA	NA	-0.120	0.914	-0.199	0.335
	PRRX1	NA	NA	-2.990	2.77E-08	-3.377	2.32E-15
	TCF4	-0.639	0.999	0.364	0.560	-0.455	0.091
	FOXC2	0.437	0.786	-0.930	0.390	-1.278	0.322
	SIX1	0.024	0.999	0.160	0.844	-0.419	0.023

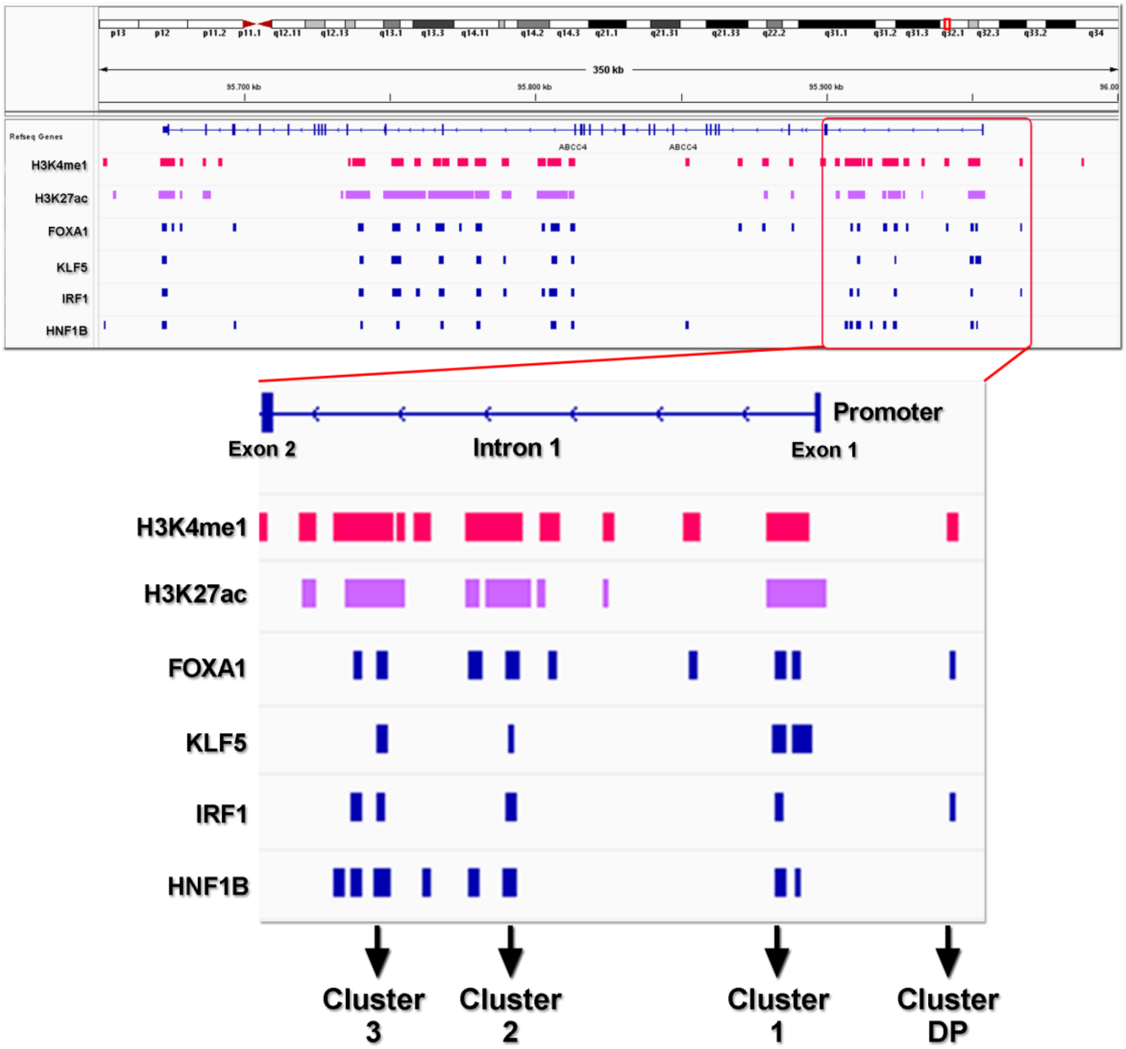

Figure S1. Identification of *ABCC4* regulatory clusters in PDAC. ChIP-seq peaks reported in CFPAC1 for poised/active *cis*-regulatory elements H3K4me1/H3K27ac and TF associated with *ABCC4*. Data sourced from GSE64557 (Diaferia et al., 2016), with tracks visualized using Integrative Genome Browser (IGV) software.

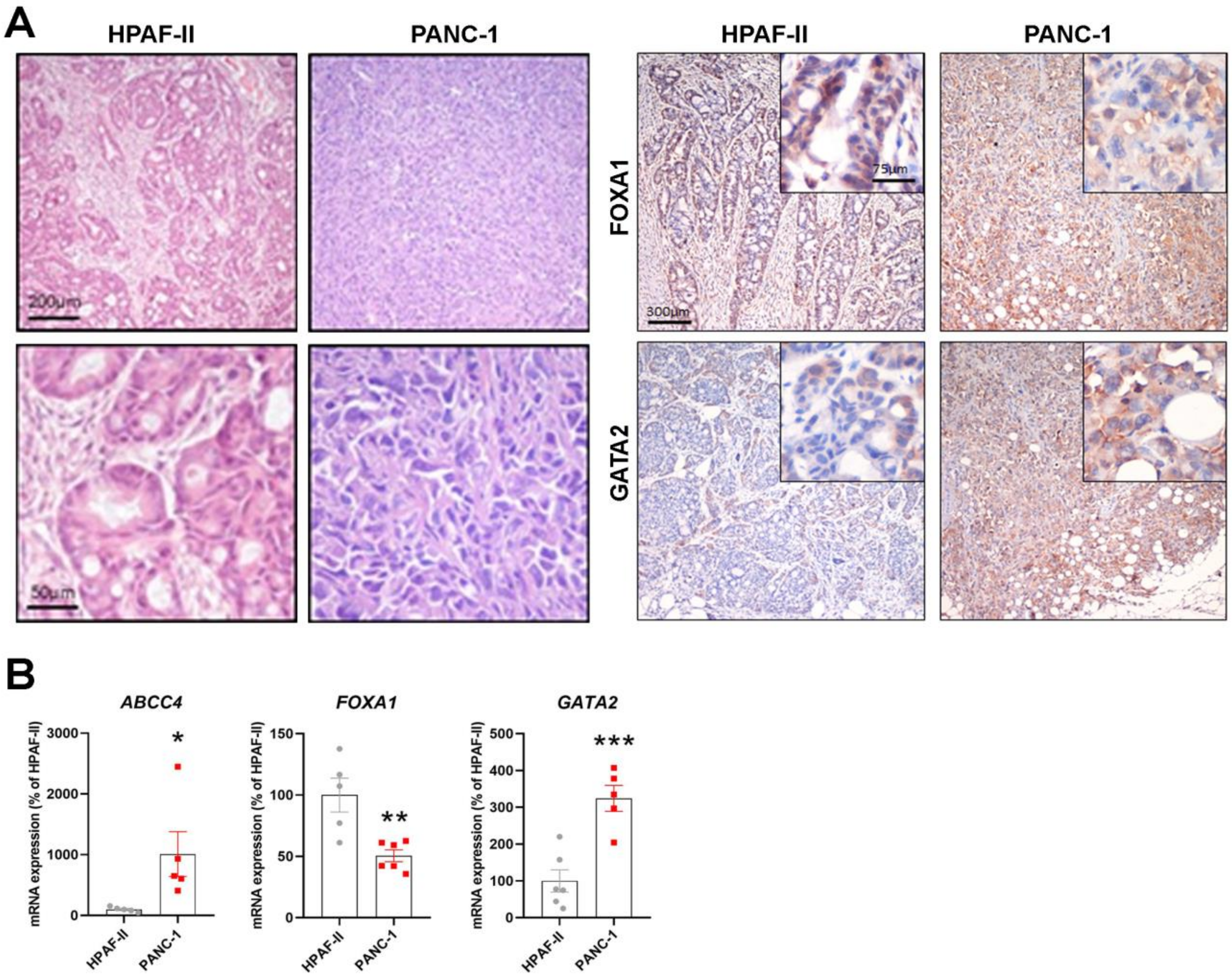

Figure S2. Characterization of *FOXA1* and *GATA2* levels in HPAF-II and PANC-1 xenografts. A) left: Histology of HPAF-II and PANC-1 xenografts (H&E); right: FOXA1 and GATA2 immunostaining in HPAF-II and PANC-1 xenografts. **B)** *ABCC4*, *FOXA1*, and *GATA2* transcript levels in HPAF-II *vs.* PANC-1 xenografts, determined by qPCR. Data is presented as mean ± SEM (N=5-6). Student's *t* test, * p<0.05, ** p<0.01, *** p<0.001.

Supplemental Materials and Methods

Immunohistochemistry (IHC) and histological staining

Formalin-fixed, paraffin-embedded xenograft sections were dewaxed in xylene, rehydrated through graded ethanols, and then incubated for 20 minutes at room temperature with 10 % H_2O_2 in ethanol 70% to quench endogenous peroxidase activity. Antigen retrieval was achieved by boiling tissue sections for 50 minutes in sodium citrate buffer (10 mM; pH 6). Subsequently, the slides were incubated in 2.5% albumin (Sigma-Aldrich) in PBS for 1 hour and allowed to react

with the primary antibodies overnight at 4 °C. After washing with PBS, the sections were incubated for 1 hour at room temperature with biotin-conjugated secondary antibodies (Vector Labs), and the avidin/biotin peroxidase complex (Vectastain Elite ABC kit; Vector Labs). Primary and secondary antibodies were used at 1/100 and 1/400 dilutions, respectively. Anti-FOXA1 (sc-514695) and anti-GATA2 (sc-267) were purchased from Santa Cruz Biotechnology, Inc.

RT-qPCR

Total RNA was extracted from PANC-1 and HPAF-II xenografts using QuickZol (Kalium Technologies) following the manufacturer's protocol. Five hundred nanograms (500 ng) of RNA were treated with DNAseI (Invitrogen) and were reverse-transcribed in a 20 µL reaction using M-MLV reverse transcriptase (Promega) and random hexameres (Biodynamics). PCR primers were designed for the specific amplification of human *ABCC4*, *FOXA1*, and *GATA2* transcripts (sequences are listed on Table S1 in the Supplemental Information). Each sample was assayed in duplicate using 4 pmol of each primer, 1X HOT FIREPol' EvaGreen qPCR Mix Plus (Solis Biodyne), and 2-20 ng of cDNA in a total volume of 13 µL. Amplification was carried out in an ABI PRISM 7500 sequence detection system (Applied Biosystems). mRNA levels for each gene were normalized to the reference gene *HPRT1*. Results are presented as % changes calculated by the ratios of normalized target genes of each group.

Primer sequences for RT-PCR

Gene	Genebank	Forward	Reverse	Product (bp)	T
ABCC4	NM_005845	GGACAAAGACAACTGGTGTGCC	AATGGTTAGCACGGTGCAGTGG	156	60
FOXA1	NM_004496	GCTACTACGCAGACACGCAG	TAGGACATGTTGAAGGACGCC	154	55
GATA2	NM_001145661	CTACCCCCAGCTCCTACCCT	CTGGGTGCAGACGGCAA	156	55
HPRT1	NM_000194	CGTGATTAGCGATGATGAACCA	CCCCTTGAGCACACAGAGG	187	60
